# Relationship between single and multiple perpetrator rape perpetration in South Africa: A comparison of risk factors in a population-based sample

**DOI:** 10.1186/s12889-015-1889-9

**Published:** 2015-07-07

**Authors:** Jewkes R, Sikweyiya Y, Dunkle K, Morrell R

**Affiliations:** Gender & Health Research Unit, Medical Research Council and School of Public Health, University of the Witwatersrand, Private Bag X385, Pretoria, 0001, South Africa; Gender & Health Research Unit, Medical Research Council, Pretoria, Gauteng South Africa; Research Office, University of Cape Town, Cape Town, Western Cape South Africa

**Keywords:** Rape, Epidemiology, Multiple perpetrator, Gang, Risk factors,

## Abstract

**Background:**

Studies of rape of women seldom distinguish between men’s participation in acts of single and multiple perpetrator rape. Multiple perpetrator rape (MPR) occurs globally with serious consequences for women. In South Africa it is a cultural practice with defined circumstances in which it commonly occurs. Prevention requires an understanding of whether it is a context specific intensification of single perpetrator rape, or a distinctly different practice of different men. This paper aims to address this question.

**Methods:**

We conducted a cross-sectional household study with a multi-stage, randomly selected sample of 1686 men aged 18–49 who completed a questionnaire administered using an Audio-enhanced Personal Digital Assistant. We attempted to fit an ordered logistic regression model for factors associated with rape perpetration.

**Results:**

27.6 % of men had raped and 8.8 % had perpetrated multiple perpetrator rape (MPR). Thus 31.9 % of men who had ever raped had done so with other perpetrators. An ordered regression model was fitted, showing that the same associated factors, albeit at higher prevalence, are associated with SPR and MPR.

**Conclusions:**

Multiple perpetrator rape appears as an intensified form of single perpetrator rape, rather than a different form of rape. Prevention approaches need to be mainstreamed among young men.

## Background

Multiple perpetrator rape (MPR), that is sex coerced by two or more perpetrators, is highly prevalent in South Africa [1, 2]. Among adult men in the Eastern Cape and KwaZulu-Natal, 9 % had had sex in an act of multiple perpetrator rape and a further 11 % had been involved in another capacity without sexual intercourse [1]. Historically multiple perpetrator rape in South Africa has been given attention as an activity of township criminal gangs [3, 4], as it has in other settings [5]. This is a context that contrasts somewhat with the informal male groupings which are commonly described in narratives of South African qualitative and ethnographic research among young men [6–8]. The former setting places multiple perpetrator rape in a socially distanced, criminal arena and yet the latter research suggests that both single perpetration rape (SPR) and MPR are rooted systemically in the prevailing gender order and specifically in constructions of masculinity amongst Black South African men.

There are a number of colloquial names for MPR including jackrolling, streamlining, istimela and so forth. Several authors have written about the cultural category of ‘streamlining’ from the rural Eastern Cape [6] [9]. They describe MPR as a form of entertainment and means of imposing gender discipline wielded by young (frequently under-occupied) men. It is very conspicuously steeped in ideas of gender hierarchy and male entitlement to use this extreme form of sexual punishment as a way of imposing a highly unequal gender order. Women and girls are expected to be sexually modest, as well as submissive to men, which includes agreeing to their sexual propositions. Streamlining is used as a way of punishing perceived transgressions of or resistance to this order. Whilst victims may be selected at random, women who are perceived to be ‘promiscuous’, those who become very drunk in public and those seen as ‘snobbish’ (rejecting the sexual advances of one or more perpetrators) are vulnerable to becoming victims. In the case of ‘promiscuous’ women, the perpetrator group often includes their male partners and the rape particularly occurs if one discovers that she has another partner [6, 7, 9]. Streamlining as entertainment often occurs when a man selects a girlfriend who he no longer likes or respects and tricks her into being in a situation where he invites his friends to rape her [6, 9, 10].

The prevalence of all forms of violence in South Africa is high and is historically linked to the social engineering and consequences of colonialism and apartheid [11, 12]. Men are much more likely to be involved in violence, both as victims and perpetrators which suggests a strong gendered link between masculinity and violence [13]. Violence is often sexualised, being expressed as rape in both homophobic and misogynistic settings. Violent behaviour is systemic and therefore learned and often legitimated [6]. In order to conceptualize the links between individual acts and social forces, Connell [14] has proposed the idea of a gender order which serves as a conceptual framework for understanding gender inequality and gender based violence. When read with analyses of South African post-apartheid society which shows the persistence of strong patterns of racial inequality and cultural continuity, it is possible to offer an explanation for the race and gender patterns associated with MPR and SPR.

Masculine identities are particularly influenced in boyhood by three institutions, the family, schools and gangs. It is in these settings that misogynism and gender inequality is learned, violence experienced and legitimated. In South Africa, the migrant labour system and apartheid’s Bantustan policy had a devastating effect on family, producing families featuring impoverished, single-headed households, grim living conditions and high levels of violence against children [15]. In schools, authoritarian pedagogies have resulted in widespread bullying, sexual predation on female students and corporal punishment [16–18]. Many boys, particularly in rural and working class settings, join gangs in their early teenage years and these organisations frequently demand demonstrations of misogyny and violence to secure membership and prestige [19]. By the time men become adults, many have experienced multiple forms of violence and regard it as an acceptable form of expression or conflict resolution. Violence against women is legitimated by the presence of discourses that naturalise a gender hierarchy, placing men as superior to women, and placing with men the right and sometimes obligation to discipline women [20–23]. These discourses are influenced by patriarchal values that particularly find expression amongst Africans where they are defended as ‘part of our culture’. Masculinities are influenced by South Africa’s raced past and current gender practices still reflect a history of raced inequality.

In South Africa, multiple rape is associated with young men (as elsewhere [24]), generally not working (including still in education) and an act associated with ‘proving masculinity’ [25]. Most of the research on streamlining suggests that this is sub-culturally accepted, or taken for granted, as a boyish activity, [1, 26] rather than being seen as an extreme, heinous, offence. This contrasts markedly with its impact on women as it can cause very severe injury, even death, and is highly stigmatising and defiling [6, 9]. Indeed there are few accounts from women’s perspectives of streamlining, in contrast to the growing literature of interviews with men [6].

If multiple perpetrator rape in South Africa appears to be a relatively common practice of male youth, it is less clear what its relationship is to single perpetrator rape. There is some evidence that men may engage in both forms of rape, given the commonness of raping on multiple occasions [1, 10]. In so doing they display behaviour described in other countries [24, 27]. Although globally research on rape perpetration in large population-based samples is uncommon, the best available multi-country dataset, the UN Multi-Country Study of Men and Violence in Asia and the Pacific [28], is directly comparable with South African data. Among the nine sites in six countries (Bangladesh, China, Cambodia, Indonesia, Papua New Guinea and Sri Lanka), any rape had been perpetrated by between 9 (urban Bangladesh) - 61 % (Papua New Guinea) of men [29] and between 5.7 % (Rural Indonesia) and 25 % (Cambodia) of men who had ever raped a woman had undertaken MPR [29]. However, notwithstanding the overlap, it is unclear whether MPR should be viewed as a context specific intensification of SPR or whether they are distinctly different practices of different men. The aim of this paper is to determine whether MPR is best understood as a distinctly different act from SPR perpetrated by men with different drivers, or whether it is best understood as an intensification of single perpetrator rape and fueled by the same drivers. This will be explored by fitting models of characteristics of men who reported perpetrating multiple perpetrator rape, single perpetrator rape and those who had never raped. The variables examined for the models were guided by the findings of the UN Multi-Country Study which were that poverty (no high school and food insecurity), personal history of victimisation (especially in childhood sexual and emotional abuse and homophobic violence), low empathy, alcohol misuse, masculinities emphasising heterosexual performance (indicated by having many partners and transactional sex), dominance over women (use of physical IPV), and participation in gangs, having weapons and drug use were all associated with engaging in MPR [29]. Although our questionnaire did not always have the same variables and included some additional measures that are known to be potentially associated with rape (such as dimensions of psychopathy [30]) which were not in the UN Multi-country Study’s dataset.

## Methods

The study was undertaken in 2008 in three districts in the Eastern Cape and KwaZulu-Natal provinces of South Africa. These form a contiguous area, and include rural areas with communally-owned land under traditional leadership, as well as commercial farms, small towns, villages, and a city, inhabited by people of all South African racial groups, several ethnic groups (predominantly Xhosa and Zulu) and socio-economic backgrounds.

The sample used a two stage proportionate stratified design to identify a representative sample of men aged 18–49 years living in the three districts. Using the 2001 census as the primary sampling frame, 222 census enumeration areas (EAs) were selected as the primary sampling unit, stratified by district and with numbers proportionate to district population size. The sample was drawn by Statistics South Africa. In order to avoid problems caused by outdated household listings, the households in each EA were mapped by the survey team and twenty were systematically selected. In each household one eligible man was randomly selected to take part in the interview. Men were eligible for the study if they were aged 18–49 years and had slept there the night before.

Of the 222 selected EAs, two (0.9 %) had no homes, and in five (2.3 %) we could not interview because permission from the local political gatekeepers was declined (1) or we could not access any eligible home after multiple visits at different times of day (4). In all the latter EAs, we established that many households were ineligible due to age or absence of a man. We completed interviews in 215 of 220 eligible EAs (97.7 %). We sampled a total of 4473 visiting points. Of these, 1353 (37.1 %) were found to contain no eligible man, 2298 (51.4 %) contained at least 1 eligible man, and 822 (18.4 %) could not be rostered for eligibility after a minimum of 3 attempts at contact. We completed interviews in 1737 of 2298 (75.6 %) enumerated and eligible households.

Interviews were conducted in isiXhosa or isiZulu or English with data collected using self-completion on APDAs (Audio-enhanced Personal Digital Assistants), thus participants could hear and read each question and its response options. Interviews took 45–60 min to complete. Only one participant was unable to do this and he asked the fieldworker to enter his responses. During interviews the fieldworkers were in the room to help if needed. Interviews were conducted in private and family members were not around. The confidentiality was ultimately assured through self-completion.

### Measurement of rape

Rape perpetration was assessed using seven questions developed for the study and validated through cognitive interviewing, none of which actually used the word ‘rape’ [31]. They were modifications of those used previously in the Eastern Cape [10]. A typical item was “How many times have you slept with a woman or girl when she didn’t consent to sex or after you forced her?” The questions additionally asked about having forced a (former) girlfriend or wife into sex, having forced a woman who was not a girlfriend or wife into sex and having sex with a woman who was too drunk to consent. Two questions assessed multiple perpetrator rape perpetration: How many times have you and other men had sex with a woman at the same time when she didn’t consent to sex or you forced her? How many times have you and other men had sex with a woman at the same time when she was too drunk to stop you? Never, once and more than once were the response options. In this paper we consider a man to have perpetrated a multiple perpetrator rape if he had indicated he had done so in responses to either of the two questions, otherwise he was classified as having perpetrated a single perpetrator rape if he answered affirmatively to any of the five rape questions that did not specify multiple perpetrators, or having never raped, if he responded in the negative to all rape questions.

### Other variables

The questionnaire included categorical variables measuring age and income. Questions on men’s childhoods included items on whether and how often their father was at home and mother’s level of schooling.

Data on adverse experiences before the age of 18 were collected using a locally modified version of the short form of the Childhood Trauma Questionnaire [32, 33]. We assessed five dimensions of adversity: emotional neglect, emotional abuse, physical neglect/hardship, physical abuse and sexual abuse using a four point response scale (never, sometimes, often and very often) (Cronbach’s alpha 0.79). A typical question was “before I reached 18 one or both of my parents were too drunk to take care of me”. Men were asked if they had ever been raped by a man (“persuaded or forced to have sex when you did not want to”).

Data were collected on two dimensions of psychopathy. Blame externalisation and Machiavellian egocentricity are two core affective and interpersonal deficits of psychopathy [34]. Blame externalisation is a perception of the world as hostile and others being at fault for one’s problems and Machiavellian egocentricity is a measure of narcissism and ruthless attitudes towards others [34]. Thirteen questions on Machiavellian Egocentricity and Blame Externalisation sub-scales of the Psychopathic Personality Inventory- Revised (PPI-I) were included. The Cronbach’s alpha for the scales together were 0.83. A typical item on the Machiavellian Egocentricity sub-scale was “I get mad if I don’t receive special favours I deserve” and on the Blame Externalisation sub-scale was “I have often been betrayed by people I trust”. Each has a 4 level response option (false, mostly false, mostly true, true). We dichotomised the scales and present the proportion scoring in the upper third of the scale versus the lower two-thirds. For blame externalisation 28.4 % were in the upper third and for Machiavellian egocentricity 18.5 % were in the upper third. These were adapted and reproduced by special permission of the Publisher Psychological Assessment Resources, Inc., 16204 North Florida Avenue, Lutz, Florida 33549, from the Psychopathic Personality Inventory- Revised by Scott O. Lilienfield, Ph.D., Copyright 2005 by PAR, Inc. Further reproduction is prohibited without permission of PAR, Inc.

We asked four items to measure empathy, adapted from Abbey et al. [35] (Cronbach’s alpha 0.80). A typical item was “I am often touched by things that I see happen”. These had a five point response scale (doesn’t describe me well – describes me well). Perceptions of life success were assessed with the following question: “If you compare your life circumstances overall now with those of the people you grew up with, would you say you have done much better for yourself, somewhat better, the same, less well, much less well?”

Attitudes towards gender relations were measured using 10 items from the Gender Equitable Men scale [36] (Cronbach’s alpha 0.78). A typical item is “There are times when a woman deserves to be beaten”. A high score denotes more equitable attitudes. Rape myths were measured on a 4-item scale where a higher score denoted more myth belief. A typical item was “in some rape cases women actually want it to happen” (Cronbach’s alpha was 0.76).

The school bullying perpetration score was an 8-item scale used to measure experiences with sexual bullying at school with four level response options (never, sometimes, often and very often) (Cronbach’s alpha 0.76). These questions were developed for the study. A typical item was “My school friends and I were a group and we would put pressure on a girl to date one of us until she agreed”.

We asked 11 items about lifetime experiences of participation in crime. These were modified for the local context from Tremblay et al. [37] who developed them as a measure of delinquency in childhood. Eight of the items related to theft (Cronbach’s alpha 0.81) and a typical item was “how often have you stolen an animal from someone?” The response options were never, once, 2–3 times and more often. Men were also asked about weapons ownership and possession and arrests.

Practices of gender relations were measured through questions about number of sexual partners, and about transactional sex with women, defined as sex that was primarily motivated by a desire for material gain on the part of the woman. This was defined as providing food, cosmetics, clothes, transportation, items for children or family, school fees, somewhere to sleep, handyman work, or cash [38]. Men were asked their age at first sex, and about lifetime perpetration of physical intimate partner violence, using the modified WHO violence against women instrument [39]. Specific acts of violence were asked in five items ranging from slapping to threats with or use of a weapon.

Recent alcohol consumption in the past 12 months was assessed through a question on frequency of having 5 or more drinks per drinking day. Drug use was assessed through a question on how often the man had smoked dagga (cannabis) in the past 12 months. This drug was selected as it grows well locally and is cheap and so is most widely used, and most men who use other drugs use this too. Men were asked if they had ever been in a gang.

### Ethical issues

The men were informed about the study, given an information sheet and signed informed consent. As an incentive, they were given R25 (US $3.2) for the interview and those who gave a blood sample for HIV testing were given a further R25 (data not discussed in this paper). Consent for the interview, completion of the interview and the request for blood for HIV testing were performed in sequential stages so that a man who might decline to give blood for HIV could still agree to the interview. It was unlikely that being asked for blood was a major deterrent to the interview as very few of those eligible who were asked for an interview declined, but many men did decline the blood sample. Since the questionnaire asked men to disclose a range of criminal acts and South African law does not privilege research data, interviews were conducted anonymously. No identifying details of the men or their households were kept after the interview and the consent forms could not be linked. Ethics approval was given by the South African Medical Research Council’s Ethics Committee.

### Data analysis

The study design provided a self-weighted sample. Data files were collated and analyses were carried out using Stata 12.0. All procedures took into account the two stage structure of the dataset, with stratification by district and the EAs as clusters. The distribution of social and demographic characteristics, childhood experiences, experience of abuse and rape victimisation, attitudes, psychological measures, aspects of gender relations, substance use and engagement with other violent and anti-social behaviour variables by rape perpetration status were summarised as percentages (or means), with 95 % confidence limits calculated using standard methods for estimating confidence intervals from complex multistage sample surveys (Taylor linearization). Pearson’s chi was used to test associations between categorical variables. Random effects ordered regression models were used to assess bi-variable associations between rape categories and continuous variables.

No efforts were made to replace missing data. However, we tested the impact of this on our findings through an analysis where we imputed values of variables that were missing provided there was some information to guide this. For some scale or score-based variables we have imputed a value based on the information from other variables in the scale/score. Where the range of possible values for the missing variables were limited and the value of the missing derived variable could be reliably imputed we did this. In other cases it was imputed based on the most probable values (taking into account available data). Where more than two variables were missing from a scale or score we did not impute but kept the value at missing. This analysis with missing data imputed did not change the overall results and is not presented.

Ordered logistic regression was used to investigate the association between the rape categories and variables describing the men’s characteristics. To account for clustering of men within EAs, we used a random effects ordered regression model. All the variables shown in the preceding tables were candidates for inclusion in the model. Variables were entered and backwards elimination was performed with variables retained at a conservative p < 0.2. The final model was then derived with variables retained at p ≤ 0.05. We tested for interactions between retained variables and found none. We tested the parallel regression assumption of the model, and examined it for each covarate and found it not violated (Brand test, p = 0.141).

## Results

Among these men, 27.6 % (466/1686) had ever raped and 8.8 % (149/1686) had perpetrated multiple perpetrator rape, thus 31.9 % (149/466) of men who had ever raped had done so with multiple perpetrators. They were mostly a sample of young men, with half aged 18–24 years. There were no age differences between the groups of men by their rape perpetration status. They did differ by socio-economic status, with more men who had raped earning over R500 (US $ 64) per month than those who had not, and the largest proportion in this earning group found among those who had perpetrated gang rape (30.2 % never raped v. 37.3 % SPR v. 47.6 % MPR). See Table 1.

**Table 1:** Socio-demographic and behavioural characteristics of men by rape perpetration category

	Never raped			Single perpetrator			Multiple perpetrator			
	n = 1220			n = 317			n = 149			
	%/mean	95 % CI		%/mean	95 % CI		%/mean	95 % CI		p value
Age: 18-24	52.4			50.2			49.0			0.405
25-34	29.9			33.8			36.2			
35-49	17.7			16.1			14.8			
Earning > R500 per month	30.2	26.9	33.6	37.3	31.7	42.9	47.6	39.9	55.3	<0.0001
Mother's education: none	25.4			17.3			13.6			<0.0001
some	68.0			71.6			66.7			
completed school	6.6			11.1			19.7			
As a child, father rarely/never home	65.2	62.4	68.1	73.1	67.7	78.4	72.1	64.4	79.9	0.024
Childhood trauma scale	18.48	18.18	18.78	20.39	19.84	20.94	22.56	21.23	23.90	<0.0001
Forced into sex by a man	6.3	4.9	7.7	16.2	11.7	20.7	19.5	13.6	25.4	<0.0001
Empathy score	14.21	13.75	14.67	13.41	12.69	14.13	13.19	12.17	14.21	0.01
Life circumstances less good than peers	16.9	14.6	19.1	26.0	21.1	30.9	32.6	23.8	41.3	<0.0001
Blame externalisation**	32.10	29.00	35.20	49.30	43.50	55.10	45.70	37.00	54.30	<0.0001
Machiavellian egocentricity**	13.90	11.70	16.20	29.50	24.00	34.90	33.30	25.30	41.30	<0.0001
Gender equitable men (GEM) scale	23.81	23.43	24.19	22.06	21.46	22.66	22.99	22.06	23.91	n.s.*
Rape myth beliefs	9.42	9.20	9.64	9.93	9.57	10.30	9.81	9.32	10.29	0.016
Bullying at school: none	36.9			13.3			6.9			<0.0001
1-2 times or forms	34.6			31.2			15.1			
many times	28.6			55.5			78.1			
Theft: never	50.4			23.7			21.2			<0.0001
1-2 times	25.2			20.3			16.1			
>2 times	24.4			55.9			62.8			
Gang membership	7.0	5.2	8.8	16.2	11.7	20.6	35.0	26.6	43.4	<0.0001
Past year alcohol : high consumption	14.1	11.8	16.5	26.7	20.8	32.6	27.7	19.5	35.9	<0.0001
Past year drug use	31.6	28.4	34.8	53.0	46.8	59.3	63.1	55.1	71.2	<0.0001
First sex at age 16 or over	60.7	57.6	63.8	48.1	42.3	54.0	43.9	35.8	52.1	<0.0001
20+ lifetime sexual partners	25.3	22.6	27.9	49.5	43.9	55.2	59.7	51.8	67.6	<0.0001
Transactional sex	59.9	56.5	63.4	81.1	76.6	85.7	84.6	78.9	90.3	<0.0001
Physical intimate partner violence (IPV)	32.7	30.1	35.4	67.4	61.8	73.0	67.8	60.2	75.5	<0.0001

*p value not shown as proportional odds assumption violated

**proportion in the upper third of the distribution

The men differed in many aspects of their childhood and early years by rape category. They mostly were raised by mothers who had not completed high school, but a greater proportion of men who raped had mothers who had completed high school, and among the MPR perpetrators it was the highest (6.6 % never raped v.11.1 % SPR v.19.7 % MPR). Most of the men reported their father to have been often or always absent in their childhood and this was higher among men who raped than those who had not.

The groups of men differed in their mean score on the scale measuring trauma in childhood, with the mean score increasing across the groups from those who had never raped to those who had perpetrated multiple perpetrator rape. The proportion that had been forced into sex by a man similarly rose across the groups (6.3 % never raped v.16.2 % SPR v. 19.5 % MPR).

On examining measures of psychological variables the groups of men again were found to differ. Empathy was lower and blame externalisation was higher among men who raped, but did not differ much between the two groups of men who had raped. Machiavellian egocentricity scores rose markedly across all three groups (14.4 never raped v. 16.6 SPR v. 17.8 MPR; p < 0.0001). The groups of men did not differ in their scores on the gender equitable men scale. On rape myths, those who had raped scored higher than those who had not.

There were differences between the groups in engagement with other anti-social behaviour. There were substantial differences in the proportion who had sexually bullied at school between the groups, with the proportion who had done so many times increasing from 28.6 % among those who never raped, to 55.5 % among those who had done SPR, to 78.1 % among those who had perpetrated with others. The same pattern was seen for engagement in theft, with the proportion who had stolen two or more times ranging across the groups from 24.4 % never raped v. 55.9 % SPR v. 62.8 % MPR. The same was seen for gang membership with the proportion ranging from 7 % never raped v. 16.2 % SPRv. 35.9 % MPR.

The men differed in their substance use, with those who had raped being more likely to be heavy drinkers than those who had not. Drug use in the past year was common and rose across the three groups from 31.6 % never raped to 53.0 % SPR to 63.1 % MPR.

The men who had raped had had sex for the first time at a younger age than those who had not done so, with the proportion having sex for the first time at 16 years or older falling across the three groups. There was a similar pattern with respect to partner numbers with the proportion who had had 20 or more rising across the groups from 25.3 % never raped to 49.5 % SPR to 59.7 % MPR. The men who had raped were much more likely to disclose having ever had a transactional sexual relationship or encounter. They were also more likely to have been physically violent towards a partner, but the proportion did not differ between the two rape groups.

The data analysis approach showed that an ordered regression model could be fitted to the data. Table 2 presents a multivariable ordered regression model of the variables showing trends across the three categories. The ordered regression model compares first the position of either having never raped or having just done a SPR with that of having engaged in a MPR, and then compares having done a MPR and/or a SPR with having never raped. In essence it presents variables associated with the difference between the most severe rape (MPR) and SPR/no rape, and between any rape (SPR and/or MPR) and no rape (note that there is only one set of odds ratios presented in this type of model as the odds ratios for these different positions are the same).

**Table 2:** Multivariable ordered regression model of the three rape perpetration categories, adjusted for age

	OR	95 % CI		p value
Mother's education: none	1.00			
some	1.33	0.87	2.03	0.192
completed school	2.61	1.47	4.63	0.001
Forced into sex by a man	2.04	1.35	3.08	0.001
Machiavellian egocentricity	1.57	1.14	2.17	0.006
Life circumstances less good than peers	1.56	1.10	2.23	0.014
Bullying at school: none	1.00			
1-2 times or forms	1.65	1.05	2.61	0.03
many times	3.14	2.05	4.81	<0.0001
Theft: never	1.00			
1-2 times	1.17	0.79	1.72	0.429
>2 times	1.80	1.19	2.72	0.006
Gang membership	1.76	1.15	2.68	0.009
Used drugs in the past year	1.52	1.10	2.11	0.011
20+ lifetime sexual partners	1.62	1.17	2.22	0.003
Ever transactional sex	1.53	1.04	2.25	0.032
Physical intimate partner violence	1.87	1.39	2.53	<0.0001

Wald chi p < 0.0001; brant test =0.141

The variables which were associated with ordered progression across these positions were having a mother who had completed high school (which increased the odds of ever perpetration rape (SPR or MPR) by more than 2.5 times), having been forced into sex by a man (which doubled the odds of having ever perpetrated rape), Machiavellian egocentricity scores (which increased the odds of ever perpetration of rape (SPR or MPR) by over 50 %), perceiving their life circumstances to be less good than those of peers (which increased the odds of ever perpetration of rape (SPR or MPR) by over 50 %), engagement in antisocial behaviour in the forms of sexual bullying at school (which increased the odds of ever perpetration of rape (SPR or MPR) more than 3 fold among those who had engaged in 3 or more types of bullying), theft (which increased the odds of ever perpetration rape (SPR or MPR) among those who had engaged in theft twice or more as opposed to never by 80 %) and gang membership (which increased the odds of ever perpetration of rape (SPR or MPR) by about 75 %), using drugs in the past year (which increased the odds of ever perpetration of rape (SPR or MPR) by over 50 %), having had twenty or more lifetime partners (which increased the odds by over 50 %), transactional sex (which increased the odds by over 50 %) and having been physically violent to a partner (which increased the odds by almost 90 %). Examination of the proportional odds assumption test in the dataset with missing values imputed drew our attention to the fact that having been physically violent to a partner does not vary much between SPR and MPR categories, although the test for this is not significant in the main model (and thus the assumption is not violated).

## Discussion

When compared to the UN Multi-country Study on Men and Violence’s findings, the proportion of men who had ever raped in our study was well within the total range, which was from 11.1 % in Bangladesh to 60.7 % in Papua New Guinea [28, 29], however the proportion who had perpetrated MPR (8.8 %) was substantially higher in South Africa than in most of the countries of the UN Multi-Country Study (most were in the range of 1.4 % in urban Bangladesh to 5.2 % in Cambodia, with the two outliers being 6.8 % in Jayapura, in Indonesia (West Papua Province) and 14.1 % in Papua New Guinea). This provides some support for an argument that MPR in South Africa is a product of the prevailing patriarchy rather than being the exceptional behaviour of a small group of criminal men. The observation that a single set of factors is associated with the progression from no rape to any rape (SPR and/or MPR) and then from no rape/SPR to MPR, which is the interpretation of the ordered model, indicates that multiple perpetrator rape is an intensification of the phenomenon of single perpetrator rape, rather than an entirely different phenomenon. We have shown that the risk factors for multiple perpetrator rape do not differ from those for single perpetrator rape, but the men who have perpetrated multiple perpetrator rape have a higher level of exposure to them. The exception is perpetration of physical partner violence which seems to be elevated for both SPR and MPR groups and not greatly differing between these.

The relationship between SPR and MPR in this South African dataset is different from that found in the six countries of Asia and the Pacific. In all of those countries there were differences in the risk factors for SPR and for MPR [29], which were not found in South Africa, and to the extent that there were differences in analysis between this paper and that from Asia and the Pacific the direction of expected impact would be towards seeing greater differences in SPR and MPR in South Africa (which were not seen). The factors associated with rape in our study were having a high social status home background (more educated mothers); a personal victimisation history of having been forced into sex by a man; having a higher score on Machiavellian egocentricity and being more likely to perceive their life circumstances were less good than their peers; having engaged in other anti-social behaviour including bullying at school and theft, and current drug use (cannabis). Further, having engaged in practices demonstrating an exaggerated and dominant (hetero) sexuality, having more partners, transactional sex and being more likely to have been physically violent to a partner. There are many similarities between these factors and those found in Asia and the Pacific. Indeed only two differences are striking. In Asia and the Pacific, multiple perpetrator rape was associated with socially marginalised circumstances of greater poverty (food insecurity) and gang membership, in contrast in South Africa gang membership was associated with all types of rape and raping was associated with relative social advantage (having a more educated mother) within a context largely of poverty. Since the latter is seen as an indicator of (highly gendered) sexual entitlement, and all rape is predominantly motivated by sexual entitlement, this may partially explain why rape is so common in South Africa.

The findings of this analysis provide important additional evidence to the growing body of research on drivers of rape perpetration. They suggest that in countries studied in Asia and the Pacific, SPR and MPR may be somewhat different phenomena, but in South Africa they are not different. They may be perpetrated in South Africa by men who are more heavily exposed to a set of risk factors and past behaviours, but these risk factors and behaviours are not inherently different for those perpetrating MPR and SPR. Given that rape in South Africa has been perpetrated by over one in four men, an argument may be advanced that rape of both types are culturally embedded features of masculinity, rather than exceptional practices of a small group of dangerous men. The implications here are that interventions to prevent both SPR and MPR need to be applied to men in the general population.

In this article we argue that rape (both MPR and SPR) needs to be understood within a broader gendered context in which particular constructions of masculinity are embedded and as existing along a continuum of violent sexual acts perpetrated against women. Men who commit either MPR or SPR can be identified with a cluster of particularly violent and anti-social activities and attitudes, although the former group are placed further along the continuum of violence against women than men who commit SPR.

The study was cross-sectional and so it is impossible to be sure of the temporal sequence of many of the factors associated with raping. It is possible that attitudes towards women and gender relations were formed as a post-hoc cognitive justification of the act. However given that our formative research showed that many men did not think that they had ‘raped’ when disclosing acts of sexual coercion of women, this is unlikely to be a major consideration [31]. Childhood factors are likely to have preceded the first act of rape and definitionally preceded rape reported in the previous year. Psychoanalytic literature on personality disorders suggests that psychopathy is likely to develop from early childhood experiences and may be genetically influenced [40–42]. It seems likely that exposure to sexual abuse and other trauma in childhood generates insecurity and a perception of being ‘hard done by’, which in turn generates an exaggerated sense of entitlement and lack of respect for the concerns of others.

The anti-social behaviours – engagement in theft and bullying at school – may have co-occurred with multiple perpetrator rape perpetration. It is possible that they stem from a common underlying tendency to so engage, and longitudinal research on school bullying from elsewhere shows that it is highly predictive of later offending [43, 44]. Drug use may similarly be part of this cluster of practices. Analysis of qualitative accounts of circumstances in which multiple perpetrator rape occurs shows many similarities with the dynamics of bullying [6, 9] [10]. Given the young age of the men involved in multiple perpetrator rape, it may be that it is best understood as an extreme form of sexual bullying. Framed in this way it suggests that avenues for prevention could draw on some of the strategies that have been developed to counter bullying [45].

The main strength of this study is that it involved a large randomly selected sample of adult men from the general population, the questionnaire included items to capture all the main variables previously found to be associated with rape and the survey had a good response rate. The findings should be generalisable. Under-reporting of rape and other anti-social behaviours in the survey is a risk, but we hope that the confidential interview process with self-completion of the questionnaire will have minimised this. Indeed the high level of reporting of rape suggests some success. The response rate was good, but non-respondent bias remains a risk. The men interviewed were younger than men in the population over all and there was a high refusal rate in areas where the population was predominantly White (48.6 %), but analysis showing weighted prevalence adjusted to the Provincial age-distribution estimates was very similar to the unweighted prevalence [1].

## Conclusions

This study has advanced understanding of rape perpetration through suggesting that in South Africa multiple perpetrator rape can be viewed as an intensified form of SPR, rather than a quite different form of rape, since the same associated factors, albeit at higher prevalence, are associated with the progression from not having raped, to SPR and then MPR. Rape prevention in South Africa needs to acknowledge the fact that the majority of men have been raised in circumstances of poverty and hardship and that many have personal victimisation histories in the form of sexual violation or trauma experienced from care givers. Notwithstanding this, rape perpetration is rooted in prevailing constructions of masculinity – South Africa’s hegemonic masculinity [20] – which emphasise dominance and control over women, expressed within a context of legitimised gendered violence [46, 47] and conspicuous performances of heterosexuality, both of which are epitomised in an act of MPR. Prevention of MPR, like SPR, in South Africa need to have at their heart interventions to change dominant constructions of masculinity and associated cultural practices, whilst acknowledging the importance of strengthening the criminal justice system so that more acts of rape are formally punished.
